# Supplementary material for: Changes in susceptibility of Plasmodium falciparum to antimalarial drugs in Uganda over time: 2019–2024
Source: Nat Commun. 2025 Aug 9;16:7353. doi: 10.1038/s41467-025-62810-x (PMC12335539; doi:10.1038/s41467-025-62810-x)
Supplement: Supplementary file 1 — Supplementary Information [file 41467_2025_62810_MOESM1_ESM.pdf]

Supplementary Information

Changes in susceptibility of *Plasmodium falciparum* to antimalarial drugs in Uganda over time: 2019-2024

Martin Okitwi,[1*] Stephen Orena,[1*] Patrick K. Tumwebaze,[1] Thomas Katairo,[1] Yoweri Taremwa,[1] Oswald Byaruhanga,[1] Stephen Tukwasibwe,[1] Samuel L. Nsobya,[1] Jennifer Legac,[2] Jeffrey A. Bailey,[3] Roland A. Cooper,[4] Melissa D. Conrad,[2] Philip J. Rosenthal[2**]**

Supplementary Table 1. Statistical analysis of changes in drug susceptibilities over time (2016-2024)

Drug	Total		Eastern Uganda		Northern Uganda	
	Tau	p-value	Tau	p-value	Tau	p-value
Chloroquine	-0.190	5.93e-29	-0.194	1.72e-23	0.096	8.62e-03
MDAQ	0.137	2.65e-13	0.183	1.08e-16	-0.113	2.01e-03
Piperaquine	0.041	0.018	0.118	3.32e-09	**-0.310**	**1.92e-17**
DHA (IC_{50})	**0.377**	**1.99e-102**	**0.351**	**4.78e-68**	0.116	1.47e-03
DHA (RSA)	0.014	0.675	**0.278**	**9.26e-06**	-0.010	0.790
Lumefantrine	**0.415**	**2.48e-123**	**0.359**	**5.74e-71**	0.121	9.42e-04
Mefloquine	**0.318**	**8.80e-72**	**0.342**	**4.49e-63**	0.093	0.0111
Pyronaridine	0.093	1.29e-07	0.154	2.63e-14	-0.121	9.27e-04
Quinine	0.104	1.80e-05	0.188	2.44e-12	-0.069	0.237
Pyrimethamine	-0.122	9.18e-11	-0.053	0.010	0.043	0.370

Bold type indicates significance based on Tau ≥±0.2. The Mann-Kendall non-parametric test was used to detect statistically significant monotonic trends (change over time in a consistent positive or negative direction) in drug susceptibilities with the Kendall package in R, using months as categorical variables.

Supplementary Table 2. Susceptibilities of *P. falciparum* control strains to antimalarials

	3D7 IC_{50} (nM), median (IQR)		Dd2 IC_{50} (nM), median (IQR)	
	**Eastern**	**Northern**	**Eastern**	**Northern**
**Number of assays:**	55	15	53	12
**Drug:**				
Chloroquine	10.0 (7.6 - 13.0)	9.9 (7.7 - 12.8)	376 (237.8 -464.0)	200 (158.8 - 223.4)
MDAQ	8.6 (5.6 - 10.2)	9.2 (7.7 - 10.1)	56.0 (45.5 - 72.7)	41.5 (37.1 - 50.1)
Piperaquine	6.6 (4.2 - 11.6)	5.5 (4.4 - 6.2)	9.6 (5.2 - 15.5)	7.6 (5.9 - 10.7)
DHA	1.6 (0.8 - 2.2)	2.4 (1.7 - 3.1)	1.7 (0.8 - 2.3)	1.8 (1.4 - 2.3)
Lumefantrine	3.2 (2.7 - 6.4)	3.6 (3.2 - 6.2)	1.9 (1.2 - 3.4)	2.2 (1.7 - 3.1)
Mefloquine	5.8 (3.1 - 9.2)	5.6 (4.6 - 6.5)	6.2 (3.9 - 8.8)	3.9 (2.7 - 5.8)
Pyronaridine	2.1 (1.1 - 3.7)	1.7 (1.4 - 2.5)	3.1 (2.1 - 5.8)	1.8 (1.6 - 4.8)
Quinine	46.0 (41.0 - 67.1)	46.4 (29.6 - 55.5)	322.0 (265 - 409)	151 (113 - 223)
Pyrimethamine	13.8 (6.8 - 49.5)	5.1 (1.5 - 17.8)	22500 (17000-28400)	20100 (13200-24900)

Supplementary Table 3. Prevalence of genetic polymorphisms associated with altered drug susceptibility over time at sites in eastern and northern Uganda.

Locus	Variant	Year	Eastern Uganda							Northern Uganda						
			Number or Samples				Prevalence (%)			Number or Samples				Prevalence (%)		
			N	WT	Mix	Mut	WT	Mix	Mut	N	WT	Mix	Mut	WT	Mix	Mut
PfK13	C469Y	2016	111	111	0	0	100	0	0	-	-	-	-	-	-	-
		2017	188	188	0	0	100	0	0	-	-	-	-	-	-	-
		2018	182	179	3	0	98.4	1.6	0	-	-	-	-	-	-	-
		2019	152	151	1	0	99.3	0.7	0	-	-	-	-	-	-	-
		2020	105	105	0	0	100	0	0	-	-	-	-	-	-	-
		2021	155	148	3	4	95.5	1.9	2.6	59	41	7	11	69.5	11.9	18.6
		2022	169	159	7	3	94.1	4.1	1.8	107	88	8	11	82.2	7.5	10.3
		2023	167	159	5	3	95.2	3	1.8	134	95	19	20	70.9	14.2	14.9
		2024	73	65	4	4	89	5.5	5.5	102	73	12	17	71.6	11.8	16.7
	A675V	2016	115	115	0	0	100	0	0	-	-	-	-	-	-	-
		2017	195	195	0	0	100	0	0	-	-	-	-	-	-	-
		2018	171	169	1	1	98.8	0.6	0.6	-	-	-	-	-	-	-
		2019	152	152	0	0	100	0	0	-	-	-	-	-	-	-
		2020	105	105	0	0	100	0	0	-	-	-	-	-	-	-
		2021	156	151	1	4	96.8	0.6	2.6	59	54	3	2	91.5	5.1	3.4
		2022	170	154	7	9	90.6	4.1	5.3	108	100	2	6	92.6	1.9	5.6
		2023	176	155	10	11	88.1	5.7	6.2	149	139	3	7	93.3	2	4.7
		2024	74	67	4	3	90.5	5.4	4.1	109	101	5	3	92.7	4.6	2.8
PfCRT	K76T	2016	96	63	12	21	65.6	12.5	21.9	-	-	-	-	-	-	-
		2017	151	120	22	9	79.5	14.6	6	-	-	-	-	-	-	-
		2018	157	145	8	4	92.4	5.1	2.5	-	-	-	-	-	-	-
		2019	139	133	3	3	95.7	2.2	2.2	-	-	-	-	-	-	-
		2020	93	92	0	1	98.9	0	1.1	-	-	-	-	-	-	-
		2021	140	139	1	0	99.3	0.7	0	47	46	0	1	97.9	0	2.1
		2022	108	108	0	0	100	0	0	63	63	0	0	100	0	0
		2023	125	124	1	0	99.2	0.8	0	124	124	0	0	100	0	0
		2024	73	73	0	0	100	0	0	96	95	1	0	99	1	0
PfMDR1	N86Y	2016	104	102	1	1	98.1	1	1	-	-	-	-	-	-	-
		2017	169	165	2	2	97.6	1.2	1.2	-	-	-	-	-	-	-
		2018	165	164	0	1	99.4	0	0.6	-	-	-	-	-	-	-
		2019	151	151	0	0	100	0	0	-	-	-	-	-	-	-
		2020	105	105	0	0	100	0	0	-	-	-	-	-	-	-
		2021	143	143	0	0	100	0	0	53	53	0	0	100	0	0
		2022	120	120	0	0	100	0	0	64	64	0	0	100	0	0
		2023	141	141	0	0	100	0	0	132	132	0	0	100	0	0
		2024	71	71	0	0	100	0	0	98	98	0	0	100	0	0

	Y184F	2016	101	47	27	27	46.5	26.7	26.7	-	-	-	-	-	-	-
		2017	169	58	51	60	34.3	30.2	35.5	-	-	-	-	-	-	-
		2018	174	53	73	48	30.5	42	27.6	-	-	-	-	-	-	-
		2019	150	63	34	53	42	22.7	35.3	-	-	-	-	-	-	-
		2020	105	34	27	44	32.4	25.7	41.9	-	-	-	-	-	-	-
		2021	142	50	38	54	35.2	26.8	38	53	18	9	26	34	17	49.1
		2022	123	39	42	42	31.7	34.1	34.1	74	28	18	28	37.8	24.3	37.8
		2023	159	57	42	60	35.8	26.4	37.7	150	46	29	75	30.7	19.3	50
		2024	77	31	13	33	40.3	16.9	42.9	111	33	26	52	29.7	23.4	46.8
	D1246Y	2016	105	83	11	11	79	10.5	10.5	-	-	-	-	-	-	-
		2017	166	144	11	11	86.7	6.6	6.6	-	-	-	-	-	-	-
		2018	166	150	9	7	90.4	5.4	4.2	-	-	-	-	-	-	-
		2019	147	142	4	1	96.6	2.7	0.7	-	-	-	-	-	-	-
		2020	101	96	2	3	95	2	3	-	-	-	-	-	-	-
		2021	142	129	9	4	90.8	6.3	2.8	52	52	0	0	100	0	0
		2022	119	114	4	1	95.8	3.4	0.8	77	76	0	1	98.7	0	1.3
		2023	144	134	6	4	93.1	4.2	2.8	146	143	2	1	97.9	1.4	0.7
		2024	73	68	4	1	93.2	5.5	1.4	103	103	0	0	100	0	0
PfDHFR	N51I	2016	103	0	0	103	0	0	100	-	-	-	-	-	-	-
		2017	167	0	0	167	0	0	100	-	-	-	-	-	-	-
		2018	166	0	0	166	0	0	100	-	-	-	-	-	-	-
		2019	150	0	0	150	0	0	100	-	-	-	-	-	-	-
		2020	104	0	0	104	0	0	100	-	-	-	-	-	-	-
		2021	142	0	0	142	0	0	100	53	0	0	53	0	0	100
		2022	122	1	0	121	0.8	0	99.2	75	0	0	75	0	0	100
		2023	146	0	3	143	0	2.1	97.9	145	2	2	141	1.4	1.4	97.2
		2024	75	0	0	75	0	0	100	107	0	0	107	0	0	100
	C59R	2016	103	2	4	97	1.9	3.9	94.2	-	-	-	-	-	-	-
		2017	167	0	1	166	0	0.6	99.4	-	-	-	-	-	-	-
		2018	166	3	4	159	1.8	2.4	95.8	-	-	-	-	-	-	-
		2019	150	1	4	145	0.7	2.7	96.7	-	-	-	-	-	-	-
		2020	104	4	2	98	3.8	1.9	94.2	-	-	-	-	-	-	-
		2021	142	2	3	137	1.4	2.1	96.5	53	3	1	49	5.7	1.9	92.5
		2022	122	2	4	116	1.6	3.3	95.1	75	5	2	68	6.7	2.7	90.7
		2023	146	3	5	138	2.1	3.4	94.5	145	6	5	134	4.1	3.4	92.4
		2024	75	0	0	75	0	0	100	107	4	3	100	3.7	2.8	93.5
	S108N	2016	103	0	0	103	0	0	100	-	-	-	-	-	-	-
		2017	164	0	0	164	0	0	100	-	-	-	-	-	-	-
		2018	161	0	0	161	0	0	100	-	-	-	-	-	-	-
		2019	138	0	0	138	0	0	100	-	-	-	-	-	-	-
		2020	94	0	0	94	0	0	100	-	-	-	-	-	-	-
		2021	141	0	0	141	0	0	100	49	0	0	49	0	0	100

		2022	111	1	0	110	0.9	0	99.1	70	0	0	70	0	0	100
		2023	132	0	0	132	0	0	100	130	2	2	126	1.5	1.5	96.9
		2024	72	0	0	72	0	0	100	95	0	0	95	0	0	100
	I164L	2016	101	98	3	0	97	3	0	-	-	-	-	-	-	-
		2017	165	154	9	2	93.3	5.5	1.2	-	-	-	-	-	-	-
		2018	164	147	13	4	89.6	7.9	2.4	-	-	-	-	-	-	-
		2019	146	135	4	7	92.5	2.7	4.8	-	-	-	-	-	-	-
		2020	98	96	1	1	98	1	1	-	-	-	-	-	-	-
		2021	137	125	2	10	91.2	1.5	7.3	51	48	1	2	94.1	2	3.9
		2022	106	87	12	7	82.1	11.3	6.6	66	62	3	1	93.9	4.5	1.5
		2023	123	110	7	6	89.4	5.7	4.9	141	137	0	4	97.2	0	2.8
		2024	68	64	1	3	94.1	1.5	4.4	98	92	3	3	93.9	3.1	3.1
PfDHPS	S436H/A	2016	102	99	3	0	97.1	2.9	0	-	-	-	-	-	-	-
		2017	161	148	7	6	91.9	4.3	3.7	-	-	-	-	-	-	-
		2018	168	153	11	4	91.1	6.5	2.4	-	-	-	-	-	-	-
		2019	149	141	5	3	94.6	3.4	2	-	-	-	-	-	-	-
		2020	104	95	5	4	91.3	4.8	3.8	-	-	-	-	-	-	-
		2021	143	137	3	3	95.8	2.1	2.1	48	46	1	1	95.8	2.1	2.1
		2022	118	115	2	1	97.5	1.7	0.8	71	71	0	0	100	0	0
		2023	146	135	5	6	92.5	3.4	4.1	141	140	1	0	99.3	0.7	0
		2024	72	71	1	0	98.6	1.4	0	112	112	0	0	100	0	0
	A437G	2016	102	0	0	102	0	0	100	-	-	-	-	-	-	-
		2017	155	0	0	155	0	0	100	-	-	-	-	-	-	-
		2018	169	3	1	165	1.8	0.6	97.6	-	-	-	-	-	-	-
		2019	146	0	1	145	0	0.7	99.3	-	-	-	-	-	-	-
		2020	100	0	0	100	0	0	100	-	-	-	-	-	-	-
		2021	140	2	0	138	1.4	0	98.6	49	2	3	44	4.1	6.1	89.8
		2022	118	3	1	114	2.5	0.8	96.6	71	2	3	66	2.8	4.2	93
		2023	142	0	0	142	0	0	100	150	9	6	135	6	4	90
		2024	74	2	0	72	2.7	0	97.3	115	3	3	109	2.6	2.6	94.8
	K540E	2016	106	0	0	106	0	0	100	-	-	-	-	-	-	-
		2017	169	0	1	168	0	0.6	99.4	-	-	-	-	-	-	-
		2018	167	3	3	161	1.8	1.8	96.4	-	-	-	-	-	-	-
		2019	150	1	2	147	0.7	1.3	98	-	-	-	-	-	-	-
		2020	105	2	0	103	1.9	0	98.1	-	-	-	-	-	-	-
		2021	143	3	0	140	2.1	0	97.9	54	1	2	51	1.9	3.7	94.4
		2022	120	3	0	117	2.5	0	97.5	83	5	5	73	6	6	88
		2023	145	0	0	145	0	0	100	143	8	7	128	5.6	4.9	89.5
		2024	76	3	0	73	3.9	0	96.1	115	4	3	108	3.5	2.6	93.9
	A581G	2016	109	107	1	1	98.2	0.9	0.9	-	-	-	-	-	-	-
		2017	177	175	1	1	98.9	0.6	0.6	-	-	-	-	-	-	-
		2018	176	172	3	1	97.7	1.7	0.6	-	-	-	-	-	-	-

		2019	150	148	2	0	98.7	1.3	0	-	-	-	-	-	-	-
		2020	105	101	2	2	96.2	1.9	1.9	-	-	-	-	-	-	-
		2021	145	141	3	1	97.2	2.1	0.7	56	53	1	2	94.6	1.8	3.6
		2022	132	125	6	1	94.7	4.5	0.8	81	81	0	0	100	0	0
		2023	166	157	4	5	94.6	2.4	3	152	150	1	1	98.7	0.7	0.7
		2024	77	70	7	0	90.9	9.1	0	118	118	0	0	100	0	0

Supplementary Table 4. Copy number data for genes of interest (2019-2024)

Locus	Copies in Ugandan isolates				Copies in control isolates (mean, SD, (n))			
	n	Copies (median)	Copies (maximum)	Number > 1.5 copies (%)	3D7	Dd2	V12	KH001_053_G8
pfmdr1	661	1	1.6	1 (0.2)	0.88, 0.12 (2)	1.99, 0.61 (3)	0.86, 0.16 (5)	0.88, 0.12 (8)
pfpm2	445	1	1.6	5 (1.1)	0.87, 0.11 (2)	0.83, 0.19 (5)	0.86, 0.22 (4)	2.63, 0.21 (4)
pfpm3	352	1	1.7	4 (1.1)	1.01, NA (1)	0.83, 0.15 (5)	0.95, 0.08 (4)	2.39, 0.36 (4)

Supplementary Table 5. Genotype-phenotype associations for lumefantrine.[1]

			Number of Samples Evaluated				Median IC_{50} (nM)			p-values				Prevalence over Time					
														Eastern Uganda			Northern Uganda		
Protein	Gene ID	Mutation	Total	WT	Mix	Mut	WT	Mix	Mut	Kruskal-Wallis[3]	WT v Mix	WT v Mut	Mix v Mut	Prevalence 2016-2019	Prevalence 2020-2024	p-value	Prevalence 2021-2022	Prevalence 2023-2024	p-value
K13	PF3D7_1343700	C469Y[2]	1294	1178	59	57	7.5	12.5	16.2	2.9E-09	9.2E-06	1.0E-08	0.11	0.6%	4.9%	1.1E-06	22.3%	28.8%	0.12
		A675V[2]	1245	1178	29	38	7.5	12.9	14.8	1.3E-06	4.0E-04	7.5E-07	0.39	0.3%	7.2%	2.2E-12	7.8%	7.0%	0.85
MDR1	PF3D7_0523000	F938Y	857	829	21	7	6.2	3.1	2.3	5.4E-04	1.9E-04	0.0019	0.35	8.0%	0.0%	NA	0.0%	0.0%	NA
ACS10	PF3D7_0525100	S520N	1348	93	205	1050	13.0	8.7	7.9	0.027	0.0031	8.0E-05	0.53	96.0%	93.4%	4.4E-02	88.7%	83.5%	0.16
PMPIX	PF3D7_1430200	R135I	892	811	10	71	6.0	8.5	10.4	1.9E-04	0.17	8.7E-07	0.72	0.7%	14.9%	3.3E-19	19.9%	0.0%	NA
ApiAP1	PF3D7_0613800	S256P	1227	1160	55	12	8.2	4.6	9.1	0.010	2.7E-05	0.70	0.39	7.5%	4.5%	0.030	0.0%	2.6%	NA
		N3511D	1174	698	222	254	9.3	6.5	7.8	8.2E-04	2E-06	0.045	0.016	50.3%	36.7%	3.9E-06	26.4%	26.2%	1.00
		V562I	1221	1179	40	2	7.6	17.5	11.1	7.0E-05	1.2E-07	0.55	0.31	0.2%	4.4%	1.3E-07	0.0%	10.3%	NA
		E700A	1127	1050	47	30	7.4	9.3	14.6	0.049	0.10	7.7E-04	0.10	5.8%	5.4%	0.80	15.5%	9.0%	0.15
		D911N	953	804	145	4	6.8	18.3	4.7	2.3E-23	1.1E-27	0.52	0.17	1.8%	17.3%	2.6E-17	1.2%	56.4%	2.9E-22
AAT1	PF3D7_0629500	F442L	922	880	17	25	7.3	15.0	18.4	0.0013	0.006	1.6E-04	0.59	1.3%	2.8%	0.13	6.5%	11.3%	0.44
CARL	PF3D7_0321900	M182I	934	911	21	2	7.3	19.0	2.6	1.3E-04	1.4E-06	0.051	0.038	0.2%	5.0%	2.6E-07	0.0%	2.6%	NA
		D611N	1174	1115	55	4	7.5	20.2	44.3	2.2E-10	2.1E-12	0.0091	0.11	0.2%	7.8%	9.0E-14	0.0%	13.3%	NA
MRP1	PF3D7_0112200	F1390I	1161	1128	16	17	7.6	14.6	16.8	0.028	0.017	0.0026	0.48	0.3%	2.2%	4.9E-03	3.5%	7.9%	0.39
MRP2	PF3D7_1447900	Q230R	1134	1105	14	15	7.6	17.8	19.6	0.018	0.0034	0.0064	1.00	0.0%	2.8%	NA	16.7%	3.6%	0.001
PM2	PF3D7_1408000	D440N	1130	1073	54	3	7.6	18.6	14.1	2.6E-07	6.4E-10	0.19	0.56	0.0%	6.0%	NA	2.6%	13.3%	0.001
patatin-like phospholipase, putative	PF3D7_0218600	I1478N|N1480I|N1481S	913	375	299	239	8.9	9.1	5.8	2.1E-08	0.98	4.6E-10	4.7E-09	65.0%	55.4%	0.0040	62.5%	49.8%	0.098

[1]The Kruskal-Wallis test with Benjamini-Hochberg correction for multiple comparisons was used to identify loci with different median drug susceptibilities between WT, mixed, and mutant isolates, followed by pairwise-Wilcoxon tests with Benjamini-Hochberg correction for multiple comparisons. Loci with at least 20 samples per category and significant differences between IC_{50}s for WT and mutants are shown. Statistical tests were two-tailed, and significance was considered $p \leq 0.05$.
[2]IC_{50} for samples with other K13 mutations were not included in wild-type category
[3]Adjusted using Benjamini-Hochberg correction
NA, not applicable

Supplementary Table 6. Genotype-phenotype associations for DHA.[1]

			Number of Samples Evaluated				Median IC_{50} (nM)			p-value				Prevalence over Time					
														Eastern Uganda			Northern Uganda		
Protein	Gene ID	Mutation	Total	WT	Mix	Mut	WT	Mix	Mut	Kruskal-Wallis	WT v Mix	WT v Mut	Mix v Mut	Prevalence 2016-2019	Prevalence 2020-2024	p-value	Prevalence 2021-2022	Prevalence 2023-2024	p-value
K13	PF3D7_1343700	C469Y[2]	1302	1187	59	56	2.0	3.7	3.6	6.9E-11	8.6E-08	1.0E-08	0.82	0.6%	4.9%	1.1E-06	22.3%	28.8%	0.17
		A675V[2]	1254	1187	29	38	2.0	3.6	3.8	6.9E-05	6.2E-04	3.5E-05	0.89	0.3%	7.2%	2.2E-12	7.8%	7.0%	0.85
CRT	PF3D7_0709000	M74I|N75E|K76T	1131	1086	27	18	2.2	1.5	1.1	2.2E-04	8.9E-04	1.2E-04	0.21	15.1%	0.6%	2.5E-22	0.9%	0.5%	1
		R371I	867	811	30	26	2.1	1.6	1.1	0.0030	0.0099	2.6E-04	0.13	18.1%	1.8%	8.4E-17	1.3%	0.6%	0.55
DHPS	PF3D7_0810800	S10F	1125	1107	16	2	2.2	5.0	5.7	0.0030	4.9E-05	0.085	0.62	0.0%	3.4%	NA	1.0%	0.8%	1
MDR1	PF3D7_0523000	Y500N	981	964	7	10	1.9	2.6	5.4	0.044	0.12	6.7E-04	0.085	0.0%	0.5%	NA	11.5%	9.8%	0.82
PMPIX	PF3D7_1430200	R135I	903	822	10	71	1.8	3.1	2.9	2.2E-07	0.0041	8.5E-09	0.38	0.7%	14.9%	3.3E-19	19.9%	0.0%	NA
		T545M	1247	1216	22	9	2.1	1.1	3.5	0.0052	2.6E-04	0.55	0.095	2.9%	2.4%	0.59	0.0%	0.0%	NA
ApiAP1	PF3D7_0613800	V562I	1228	1187	39	2	2.1	4.6	2.2	2.3E-05	2.3E-08	0.95	0.11	0.2%	4.4%	1.3E-07	0.0%	10.3%	NA
		D911N	962	813	145	4	1.9	3.9	1.8	1.7E-16	1.7E-20	0.55	0.095	1.8%	17.3%	2.6E-17	1.2%	56.4%	3.9E-22
		D1016N	1130	1106	23	1	2.0	3.8	3.5	0.020	9.9E-05	0.56	0.56	0.3%	3.8%	1.5E-05	1.4%	2.8%	1
CARL	PF3D7_0321900	D611N	1181	1123	54	4	2.0	4.7	2.7	1.3E-07	1.1E-10	0.30	0.30	0.2%	7.8%	9.0E-14	0.0%	13.3%	NA
PM2	PF3D7_1408000	D440N	1136	1081	52	3	2.1	4.1	4.8	1.4E-07	6.4E-10	0.055	0.54	0.0%	6.0%	NA	2.6%	13.3%	0.0011
patatin-like phospholipase, putative	PF3D7_0218600	I1478N|N1480I|N1481S	992	376	300	246	2.2	2.2	1.7	0.0018	0.38	9.3E-05	2.1E-05	65.0%	55.4%	0.0040	62.5%	49.8%	0.098

[1]The Kruskal-Wallis test with Benjamini-Hochberg correction for multiple comparisons was used to identify loci with different median drug susceptibilities between WT, mixed, and mutant isolates, followed by pairwise-Wilcoxon tests with Benjamini-Hochberg correction for multiple comparisons. Loci with at least 20 samples per category and significant differences between IC_{50}s for WT and mutants are shown. Statistical tests were two-tailed, and significance was considered $p \leq 0.05$.
[2]IC_{50} for samples with other K13 mutations were not included in wild-type category.
NA, not applicable

Supplementary Table 7. Drug susceptibilities for isolates with varied sequences at potential drug resistance alleles.

Locus	Drug	PfK13 WT		PFK13 C469Y		PfK13 A675V	
		N	IC_{50} (nM)	N	IC_{50} (nM)	N	IC_{50} (nM)
PfCARL D611	Lumefantrine	967	6.7	38	15.2	25	15.3
PfCARL 611N	Lumefantrine	2	23.6	1	54.0	1	53.2
PfMDR1 Y500	DHA	848	1.8	28	4.0	0	NA
PfMDR1 500N	DHA	6	5.5	1	8.0	0	NA

Supplementary Table 8. Genotype-phenotype associations for pyrimethamine, mefloquine, chloroquine, and MDAQ.[1]

Drug	Protein	Gene ID	Mutation	Num. Samples Evaluated				Median IC_{50} (nM)			p-value			
				Total	WT	Mix	Mut	WT	Mixed	Mut	Kruskal-Wallis	WT v Mixed	WT v Mut	Mixed v Mut
Pyrimethamine	DHFR	PF3D7_0808100	C59R	1086	19	27	1040	20260	27071	37850	4.89E-04	0.042	1.43E-05	0.0020
			I164L	1030	949	43	38	36470	53020	81346	3.20E-11	2.64E-04	9.96E-13	0.0010
	K13	PF3D7_1343700	C469Y[2]	1116	1039	43	34	38596	30518	29440	0.031	0.0046	0.0012	0.40
Mefloquine	K13	PF3D7_1343700	C469Y[2]	1275	1161	59	55	12.5	17.5	18.2	0.0012	0.0057	5.08E-05	0.27
			A675V[2]	1228	1161	29	38	12.5	22.3	18.9	0.016	0.0013	0.016	0.31
	CRT	PF3D7_0709000	M74I|N75E|K76T	1110	1070	26	14	13.0	9.7	7.4	0.011	0.0061	0.0016	0.16
			R371I	848	799	29	20	12.6	9.0	9.0	0.022	0.0054	0.0054	0.73
	DHPS	PF3D7_0810800	S10F	1103	1086	16	1	13.0	25.9	19.9	0.0045	1.09E-05	0.42	0.42
	ACS	PF3D7_0627800	I180V	1165	160	251	754	14.7	11.0	12.4	0.016	6.63E-05	0.031	0.0027
	patatin-like phospholipase, putative	PF3D7_0218600	I1478N|N1480I|N1481S	905	367	299	239	13.2	13.0	10.5	2.36E-05	8.32E-07	2.45E-07	3.44E-08
	ApiAP1	PF3D7_0613800	V562I	1202	1161	39	2	12.4	25.0	12.1	0.0026	3.0E-06	0.88	0.29
			D911N	945	797	144	4	12.0	23.3	5.5	2.8E-14	1.0E-17	0.064	0.0080
			D1016N	1108	1084	23	1	12.3	25.3	18.2	0.0044	9.36E-06	0.47	0.47
	CARL	PF3D7_0321900	M182I	926	905	20	1	12.0	30.4	11.0	7.0E-04	6.1E-07	0.80	0.18
			D611N	1159	1101	54	4	12.0	27.7	24.0	1.08E-12	1.14E-15	0.07	0.77
	PM2	PF3D7_1408000	D440N	1115	1060	52	3	12.3	23.5	16.1	9.95E-05	8.53E-08	0.48	0.48
Chloroquine	CRT	PF3D7_0709000	M74I|N75E|K76T	1209	1143	35	31	14.2	31.0	232	1.49E-22	7.06E-08	3.23E-20	4.42E-07
			Q271E	844	786	30	28	15.0	22.0	227	6.25E-15	2.22E-05	9.30E-15	2.22E-05
			R371I	942	866	36	40	14.7	21.0	227	1.49E-22	1.21E-05	4.86E-23	3.64E-10
	PMIX	PF3D7_1430200	R135I	974	894	10	70	15.3	9.6	9.6	1.24E-04	0.021	1.16E-06	0.72
	K13	PF3D7_1343700	A675V*	1331	1265	29	37	15.0	11.7	9.1	0.025	0.092	1.32E-04	0.21
MDAQ	CRT	PF3D7_0709000	M74I|N75E|K76T	1007	988	12	7	7.4	10.5	22.5	0.020	0.022	9.59E-05	0.022

[1]The Kruskal-Wallis test with Benjamini-Hochberg correction for multiple comparisons was used to identify loci with different median drug susceptibilities between WT, mixed, and mutant isolates, followed by pairwise-Wilcoxon tests with Benjamini-Hochberg correction for multiple comparisons. Loci with at least 20 samples per category and significant differences between IC_{50}s for WT and mutants are shown. Statistical tests were two-tailed, and significance was considered p ≤0.05.
[2]IC_{50} for samples with other K13 mutations were not included in wild-type category.
NA, not applicable

Supplementary Table 9. Number of samples analyzed for data illustrated in figures.

Figure 2.

Drug	N						
**Year:**	**Total**	**2019**	**2020**	**2021**	**2022**	**2023**	**2024**
**Chloroquine**	1060	76	97	198	246	307	136
**MDAQ**	1062	76	97	198	250	305	136
**Piperaquine**	1062	76	94	198	250	307	137
**DHA**	1063	76	97	198	250	306	136
**Lumefantrine**	1060	71	97	198	250	306	138
**Mefloquine**	1058	76	97	198	249	306	132
**Pyronaridine**	1057	76	90	198	250	307	136
**Quinine**	770	62	97	149	143	209	110
**Pyrimethamine**	881	76	97	173	142	279	114
**RSA survival**	439	0	0	94	147	112	86

Supplementary Figure 1.

Drug	N						
**Year:**	**Total**	**2019**	**2020**	**2021**	**2022**	**2023**	**2024**
**Chloroquine**	707	76	97	149	152	170	63
**MDAQ**	708	76	97	149	156	168	62
**Piperaquine**	708	76	94	149	156	170	63
**DHA**	710	76	97	149	156	169	63
**Lumefantrine**	706	71	97	149	156	169	64
**Mefloquine**	709	76	97	149	155	169	63
**Pyronaridine**	704	76	90	149	156	170	63
**Quinine**	625	62	97	149	143	132	42
**Pyrimethamine**	674	76	97	149	142	151	59
**RSA survival**	126	0	0	43	47	36	0

Supplementary Figure 2.

Drug	N						
**Year:**	**Total**	**2019**	**2020**	**2021**	**2022**	**2023**	**2024**
**Chloroquine**	353	0	0	49	94	137	73
**MDAQ**	354	0	0	49	94	137	74
**Piperaquine**	354	0	0	49	94	137	74
**DHA**	353	0	0	49	94	137	73
**Lumefantrine**	354	0	0	49	94	137	74
**Mefloquine**	349	0	0	49	94	137	69
**Pyronaridine**	349	0	0	49	94	137	73
**Quinine**	145	0	0	0	0	77	68
**Pyrimethamine**	207	0	0	24	0	128	55
**RSA survival**	313	0	0	51	100	76	86

Supplementary Figure 1.

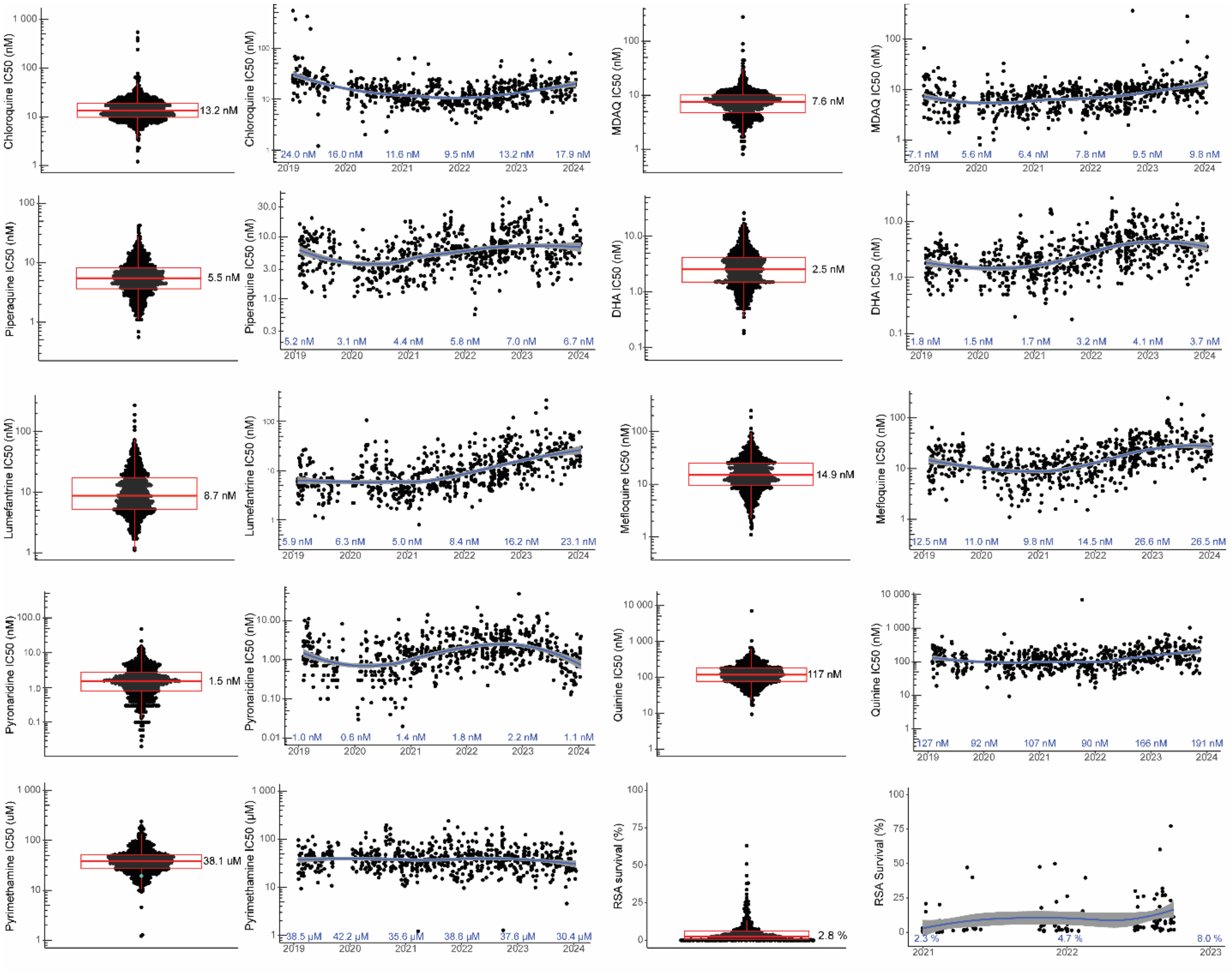

Supplementary Figure 1. Ex vivo drug susceptibilities in eastern Uganda over time. Data for samples collected in eastern Uganda are displayed as described for Figure 2. Source data are provided as a Source Data file.

Supplementary Figure 2.

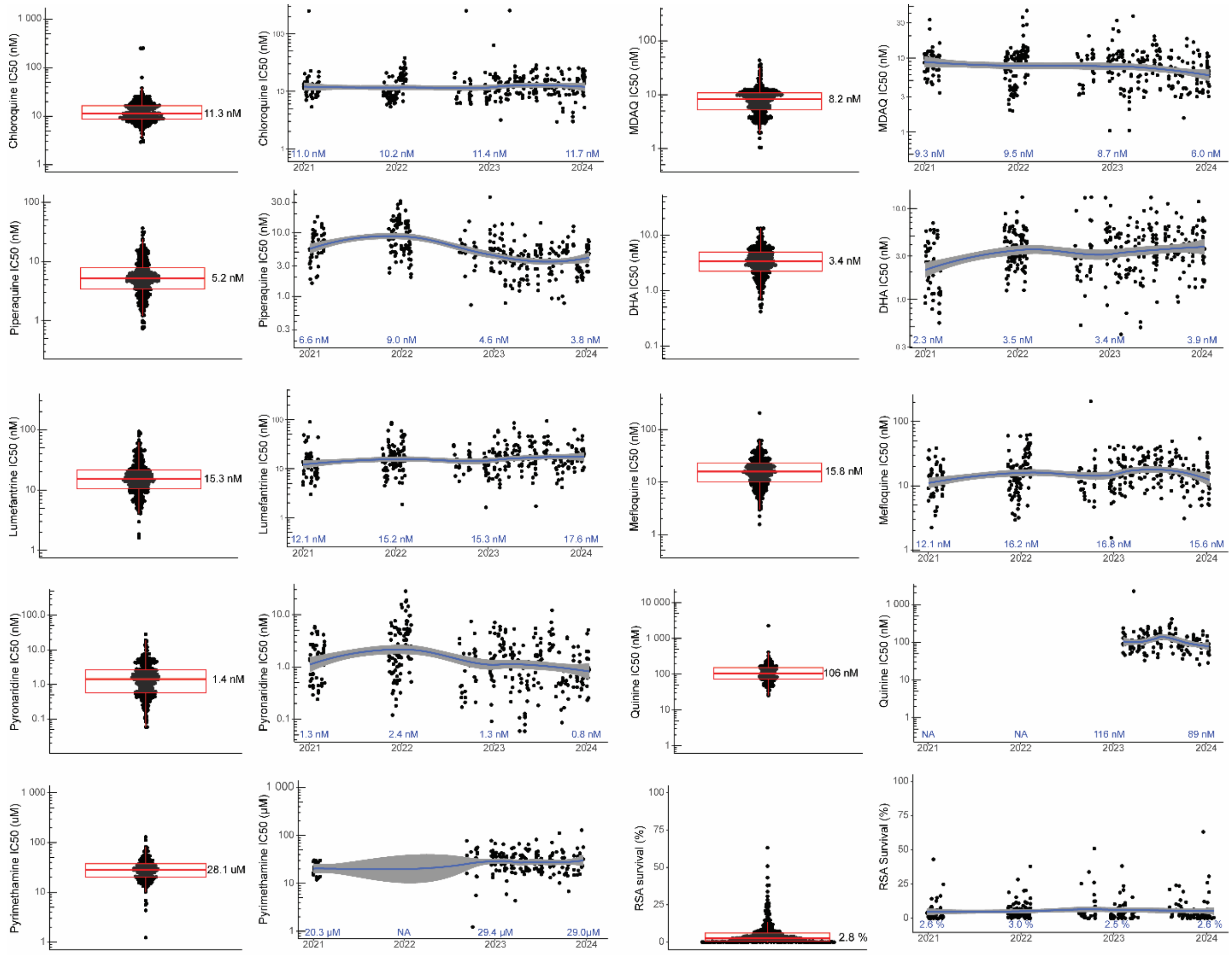

Supplementary Figure 2. Ex vivo drug susceptibilities in northern Uganda over time. Data for samples collected in northern Uganda are displayed as described for Figure 2. Source data are provided as a Source Data file.
