# Supplementary material for: Changes in susceptibility of Plasmodium falciparum to antimalarial drugs in Uganda over time: 2019–2024
Source: Nat Commun. 2025 Aug 9;16:7353. doi: 10.1038/s41467-025-62810-x (PMC12335539; doi:10.1038/s41467-025-62810-x)
Supplement: Supplementary file 2 — Reporting Summary [file 41467_2025_62810_MOESM2_ESM.pdf]

nature portfolio

Corresponding author(s): Phil Rosenthal and Melissa Conrad

Last updated by author(s): Jun 26, 2025

Reporting Summary

Nature Portfolio wishes to improve the reproducibility of the work that we publish. This form provides structure for consistency and transparency in reporting. For further information on Nature Portfolio policies, see our Editorial Policies and the Editorial Policy Checklist.

Statistics

For all statistical analyses, confirm that the following items are present in the figure legend, table legend, main text, or Methods section.

n/a	Confirmed	
☐	☒	The exact sample size (*n*) for each experimental group/condition, given as a discrete number and unit of measurement
☒	☐	A statement on whether measurements were taken from distinct samples or whether the same sample was measured repeatedly
☐	☒	The statistical test(s) used AND whether they are one- or two-sided<br>*Only common tests should be described solely by name; describe more complex techniques in the Methods section.*
☒	☐	A description of all covariates tested
☐	☒	A description of any assumptions or corrections, such as tests of normality and adjustment for multiple comparisons
☐	☒	A full description of the statistical parameters including central tendency (e.g. means) or other basic estimates (e.g. regression coefficient) AND variation (e.g. standard deviation) or associated estimates of uncertainty (e.g. confidence intervals)
☐	☒	For null hypothesis testing, the test statistic (e.g. *F*, *t*, *r*) with confidence intervals, effect sizes, degrees of freedom and *P* value noted<br>*Give P values as exact values whenever suitable.*
☒	☐	For Bayesian analysis, information on the choice of priors and Markov chain Monte Carlo settings
☒	☐	For hierarchical and complex designs, identification of the appropriate level for tests and full reporting of outcomes
☒	☐	Estimates of effect sizes (e.g. Cohen's *d*, Pearson's *r*), indicating how they were calculated

Our web collection on statistics for biologists contains articles on many of the points above.

Software and code

Policy information about availability of computer code

Data collection	IC50s were analyzed using Prism Graphpad versions 8, 9 and 10. Genotyping data was processed using MIPTools version 0.5.0
Data analysis	Data analysis was performed in RStudio 2024.09.1+394. Mann-Kendall non-parametric tests were done with the Kendall package.

For manuscripts utilizing custom algorithms or software that are central to the research but not yet described in published literature, software must be made available to editors and reviewers. We strongly encourage code deposition in a community repository (e.g. GitHub). See the Nature Portfolio guidelines for submitting code & software for further information.

Data

Policy information about availability of data

All manuscripts must include a data availability statement. This statement should provide the following information, where applicable:

- Accession codes, unique identifiers, or web links for publicly available datasets
- A description of any restrictions on data availability
- For clinical datasets or third party data, please ensure that the statement adheres to our policy

Any data will be shared with interested investigators upon request to the corresponding authors. Sequencing reads are available in the National Center for Biotechnology Information Archive (BioProject PRJNA850445).

Research involving human participants, their data, or biological material

Policy information about studies with human participants or human data. See also policy information about sex, gender (identity/presentation), and sexual orientation and race, ethnicity and racism.

Reporting on sex and gender	Sex/gender based analyses were not provided, as based on extensive prior information, we do not expect meaningful differences in malaria parasites collected from males or females. Percentages of males and females contributing isolates was reported (Table 1).
Reporting on race, ethnicity, or other socially relevant groupings	Distinctions were male vs. female based on locally acceptable criteria in Uganda.
Population characteristics	Available information is in Table 1.
Recruitment	Patients diagnosed with uncomplicated malaria during periods of observation were approached, and if they agreed they (or parents/guardians) underwent formal written consent.
Ethics oversight	The study was approved by the Makerere University School of Biomedical Sciences Research and Ethics Committee, the Uganda National Council for Science and Technology, and the University of California, San Francisco Committee on Human Research.

Note that full information on the approval of the study protocol must also be provided in the manuscript.

Field-specific reporting

Please select the one below that is the best fit for your research. If you are not sure, read the appropriate sections before making your selection.

☒ Life sciences ☐ Behavioural & social sciences ☐ Ecological, evolutionary & environmental sciences

For a reference copy of the document with all sections, see nature.com/documents/nr-reporting-summary-flat.pdf

Life sciences study design

All studies must disclose on these points even when the disclosure is negative.

Sample size	Sample size was based on convenience, entailing all samples collected and analyzed at our two laboratories, 724 in eastern Uganda and 390 in northern Uganda. This large sample afforded adequate power for many subsequent analyses.
Data exclusions	Data were excluded when, based on our analyses, parasitological or genomic data did not meet prespecified quality criteria.
Replication	Unfortunately, an inherent aspect of ex vivo assays is that they can only be performed once, shortly after the time of sample collection. Therefore, replication is not possible, but assay reliability was regularly verified based on assays using control laboratory strains of P. falciparum.
Randomization	Non applicable.
Blinding	Not applicable.

Reporting for specific materials, systems and methods

We require information from authors about some types of materials, experimental systems and methods used in many studies. Here, indicate whether each material, system or method listed is relevant to your study. If you are not sure if a list item applies to your research, read the appropriate section before selecting a response.

Materials & experimental systems

n/a	Involved in the study	
☒	☐	Antibodies
☒	☐	Eukaryotic cell lines
☒	☐	Palaeontology and archaeology
☐	☒	Animals and other organisms
☐	☒	Clinical data
☒	☐	Dual use research of concern
☒	☐	Plants

Methods

n/a	Involved in the study	
☒	☐	ChIP-seq
☒	☐	Flow cytometry
☒	☐	MRI-based neuroimaging

Animals and other research organisms

Policy information about studies involving animals; ARRIVE guidelines recommended for reporting animal research, and Sex and Gender in Research

Laboratory animals	Not applicable.
Wild animals	Not applicable.
Reporting on sex	Sex of human research participants was not considered, as risks of malaria do not notably differ between sexes and as in our many similar studies in Uganda similar numbers of males and females have consistently been recruited.
Field-collected samples	P. falciparum isolates were collected from patients presenting with uncomplicated malaria at clinics located near our laboratories.
Ethics oversight	The study was approved by the Makerere University School of Biomedical Sciences Research and Ethics Committee, the Uganda National Council for Science and Technology, and the University of California, San Francisco Committee on Human Research.

Note that full information on the approval of the study protocol must also be provided in the manuscript.

Clinical data

Policy information about clinical studies

All manuscripts should comply with the ICMJE guidelines for publication of clinical research and a completed CONSORT checklist must be included with all submissions.

Clinical trial registration	Not applicable.
Study protocol	Not applicable. This was not a clinical trial.
Data collection	We collected blood from patients diagnosed with uncomplicated malaria between July, 2019 and June, 2024 at three sites in eastern Uganda (Tororo District Hospital, Tororo District; Masafu General Hospital, Busia District; and Busiu Health Centre IV, Mbale District) and assessed drug susceptibilities at our laboratory in Tororo (Figure 1). We added collection of samples from patients with uncomplicated malaria in Agago District, in northern Uganda, first with transport of samples from Patongo Health Centre IV to our Tororo laboratory from May to July, 2021 and April to July, 2022, and since January, 2023 with samples from Patongo Health Centre IV and Dr. Ambrosoli Memorial Hospital, Kalongo assessed in a new laboratory at the hospital.
Outcomes	We measured ex vivo drug susceptibilities and sequenced the genotypes of isolates from patients presenting with malaria. Methods to obtain these outcomes are described in detail in the manuscript.

Plants

Seed stocks	Not applicable.
Novel plant genotypes	Not applicable.
Authentication	Not applicable.
